# Drivers of No-Show for Ambulatory Appendectomy at Kyabirwa Surgical Center, Jinja City, Uganda

**DOI:** 10.7759/cureus.99761

**Published:** 2025-12-21

**Authors:** Moses Bakaleke Binoga, Eve Wabule, Job Nanyiri, Arthur Emoru, Joseph Okello Damoi, Saul Kibirango, Ambrose Nuwahereza, Anna Turumanya Kalumuna, Michael Marin

**Affiliations:** 1 Surgery, Global Surgical Initiatives Inc, Kyabirwa Surgical Center, Jinja, UGA; 2 Research and Development, Global Surgical Initiatives Inc, Kyabirwa Surgical Center, Jinja, UGA; 3 Anesthesiology and Surgery, Global Surgical Initiatives Inc, Kyabirwa Surgical Center, Jinja, UGA; 4 Biomedical Engineering, Global Surgical Initiatives Inc, Kyabirwa Surgical Center, Jinja, UGA; 5 Nursing, Global Surgical Initiatives Inc, Kyabirwa Surgical Center, Jinja, UGA; 6 Surgery, Icahn School of Medicine at Mount Sinai, New York, USA

**Keywords:** ambulatory appendectomy, ambulatory surgery, kyabirwa surgical center, no-show for appendectomy, no-show for surgery

## Abstract

Background

Appendicitis is a highly prevalent acute abdominal condition that has been increasing in prevalence since 1990. Millions of cases were registered in 2024 alone, along with thousands of deaths and more than a million disability-adjusted life-years. Such outcomes are inextricably associated with complicated appendicitis that has not been surgically treated. Sadly, a significant proportion of patients diagnosed with surgically treatable appendicitis and scheduled for appendectomy do not show up for surgery, even at standalone ambulatory surgery facilities where such surgery is comparatively cheaper.

Objective

This study aimed to assess the drivers of no-shows for ambulatory appendectomy at Kyabirwa Surgical Center (KSC), Jinja, Uganda.

Method

This cross-sectional study included 36 adult patients who were diagnosed with appendicitis at Kyabirwa Surgical Center and booked for an appendectomy between 2022 and 2025. The patients were consecutively sampled and engaged at the community level through structured interviews. Clinical data were abstracted and descriptively analyzed using SPSS version 26 (IBM Corp., Armonk, USA), along with a log-binomial model for multivariate analysis in the same program.

Results

The no-show rate for ambulatory appendectomies at Kyabirwa Surgical Center was 50% (18), although 67% (24)of all patients underwent appendectomy, with one-quarter (25%, 6) receiving it at other facilities. The perception of being able to afford an appendectomy without difficulty, having personal transportation to the facility, and access to transportation in general reduce the no-show rate. However, the perceived cost of appendectomy at Kyabirwa Surgical Center was high (unaffordable), and the fear of not waking up during surgery (tomophobia) increased the no-show rate.

Conclusion

The prevention of no-shows for appendectomy among patients at KSC is concerning; only half of the patients scheduled for that surgery appear at the center. One-quarter of them underwent surgery at other facilities, while the rest did not. This patient behavior is squarely linked to individual patient characteristics, largely tomophobia, and the perceived high cost of ambulatory appendectomies. In the future, the administration and staff of Kyabirwa Surgical Center may consider preoperatively educating and sensitizing all patients scheduled for ambulatory appendectomy or any ambulatory surgery about the comparatively lower cost of their ambulatory surgical procedures at Kyabirwa Surgical Center compared to other non-ambulatory facilities. This might help alleviate negative perceptions of the affordability of surgeries, such as appendectomies, which are at the center, thereby reducing no-show prevalence.

## Introduction

Access to surgical care services has increased over the past three decades; however, the actual receipt of surgical care remains a challenge for some patients, even when they are fully eligible, largely because they fail to show up for scheduled surgeries [1, 2, 3, 4]. Patient no-shows refer to situations in which a patient booked for surgery decides not to attend the scheduled surgery and does not cancel it [4]. No-shows pose a significant challenge to the global surgical system [4], given their associations with adverse pre- and post-treatment outcomes for any surgical condition [3, 5]. However, the clinical ramifications of no-shows for surgery are disproportionate in patients with surgically correctable conditions, including complicated appendicitis.

Appendicitis is a highly prevalent acute abdominal condition that has increased in prevalence since 1990. Its incidence has generally increased by 63.55% since 1990 [6, 7], with approximately 18.6 million new appendicitis cases registered every year [6, 7]. Deaths due to acute appendicitis are uncommon, but they occur with a mortality rate of 0.358 per 100,000 people [8], which translates to approximately 29,000 deaths annually. These deaths are significantly associated with the complications of untreated appendicitis, notably perforations, abscess formation, necrosis [9,10], and peritonitis [11]. These complications are responsible for more than a million years lost due to disability, resulting in an inferior quality of life [6]. It should be known that acute appendicitis can be managed conservatively with medicines or invasively with surgery, of which the latter is considered to be the gold standard, given the high risk of recurrence with conservative treatment [12].

However, while highly effective, surgical treatment of appendicitis must be performed promptly to safeguard a good postoperative prognosis and reduce the costs associated with additional intraoperative and postoperative interventions during the management of complicated appendicitis, including percutaneous drainage [13], and abdominal drainage (prophylactic) [14]. Thus, not showing up for an appendectomy can increase the incidence of complicated appendicitis [10, 13] or its more severe form, the management of which is significantly more costly [14]. Such incremental costs can consequently prevent a patient from accessing surgical care, even at surgical facilities that have been established with the promise of providing relatively cheaper but high-quality surgeries, such as ambulatory surgery facilities.

This is because ambulatory surgery facilities still charge fees for surgery, which can increase significantly in cases of severe surgical diseases that may require more sophisticated intervention. It should be noted that appendectomies can be safely performed in ambulatory settings [15] and have been performed in recent years [16]. However, by model, ambulatory settings, especially standalone ones, such as the Kyabirwa Surgical Center, provide same-day surgery and may not cater to cases requiring prolonged admission, such as complicated appendicitis. Worryingly, no-shows for appendectomies among patients scheduled for appendectomies at such centers could be high. Between 20% and 40% of all appendectomies are conducted late [13] or when complications have already arisen [17], which is an indicator of no-shows.

Although the prevalence of no-shows for appendectomies is not well documented, there are no previously assessed drivers. Although appendicitis has been a health challenge for centuries, its treatment challenges remain [18], particularly regarding the timing of appendectomies. Most appendectomies are performed late after they have become complicated following periods of no-show for reasons not yet fully established to make them conclusive [18]. The same applies to the Ugandan context, where the only studies that have assessed no-shows or cancellations for surgery include Vahwere et al. [19] and Ogwal et al. [20], both of which focused on cancellations of surgery in general, but not patient-driven no-shows. Furthermore, they did not focus on patients diagnosed with appendicitis, and none of the studies conducted in the realm of appendicitis in Uganda, such as Farhan et al. [21], have assessed no-shows for planned appendectomy and overtly not in an ambulatory setting, such as the Kyabirwa Surgical Center (KSC).

Since its inception, the Kyabirwa Surgical Center has conducted 36 appendectomies; however, while such a milestone is laudable for Uganda’s first standalone ambulatory surgical center, it falls short of the 83 appendectomies that the facility would have conducted as of August 2025, had all patients scheduled for appendectomies shown up for the procedure. Thus, the problem of no-shows for surgery is a current and concerning issue at Kyabirwa Surgical Center; however, the magnitude and drivers of no-shows for ambulatory appendectomies at the center have not been objectively assessed before. Therefore, this study assessed the drivers of no-shows for ambulatory appendectomy at Kyabirwa Surgical Center.

## Materials and methods

Study design and area

This study employed a cross-sectional design in which a representative group of study subjects was sampled and studied at a single point in time, with both exposure and outcome variable data concurrently collected [22]. The aforementioned characteristics of the cross-sectional design made it the most suitable for this study, as it had no outcome or respondent characteristics that required a follow-up. Once a given respondent was engaged, all data required by the study were collected, including those needed to quantify prevalence, as enabled by a cross-sectional design [22]. Additionally, there was a need to establish non-causal relationships between the independent and dependent variables of the collected data, which was also enabled by a cross-sectional design [22]. This study was conducted at the Kyabirwa Surgical Center (KSC), the only standalone ambulatory surgery facility in Uganda. The center is located in Kyabirwa, a village in Budondo sub-county, Jinja City, and has been in operation since 2019, providing a wide range of quality ambulatory surgeries, including appendectomies. As a policy at the KSC, an appendectomy is scheduled only if surgery is the definitive treatment required; otherwise, a conservative route is adopted. Therefore, most appendicitis cases are treated with medication, as opposed to surgery; however, it has been noticed that a section of patients booked for appendectomies do not show up for surgery, for reasons that have not yet been studied in the Kyabirwa surgical center context. Between 2023 and 2025, the center scheduled approximately 83 patients for ambulatory appendectomies, a number that was not conducted at the center.

Study population and eligibility

The study population included adult patients (>18 years old) who had been diagnosed with appendicitis at the Kyabirwa Surgical Center and were considered for appendectomy between 2023 and 2025. This timeframe was chosen to ensure the inclusion of as many patients as possible, who were previously scheduled for surgery, thereby increasing the study's power. The study included patients who were diagnosed with appendicitis at the Kyabirwa Surgical Center because factors at a given facility of diagnosis can influence patient behavior. Therefore, to generate data that are solely representative of the Kyabirwa Surgical Center, the patients included in this study were those whose appendicitis was diagnosed at the KSC and, consequently, scheduled for surgery. The study excluded patients who experienced intense postoperative pain as a result of an appendectomy they had recently undergone or due to untreated appendicitis. Such patients were not expected to participate or provide accurate responses during the 30 to 40-minute-long structured interview. 

Sample size determination and sampling

The number of patients who were previously diagnosed with appendicitis at Kyabirwa Surgical Center and who were required to participate in this study was initially planned to be computed using the formula by Krejcie and Morgan (1970) [23], since there was a documented population size of patients who had been booked for appendectomy at KSC between 2023 and 2025. However, there was a challenge with practical access to all patients who had been booked for surgery over the previous three years. A significant number could not be reached by telephone as their numbers were unreachable. Therefore, pre-assessment of availability for interview revealed that only 36 of those previously booked for surgery, which was 43% of those scheduled for appendectomy over the previous three years, were accessible and eligible for the study. Consequently, a census was conducted, rather than a formula-based approach, as it would further reduce the probable sample size. Thus, the accessible patient population of 36 was included and sampled in a process that commenced with obtaining a list of their names and telephone contacts from the facility clinic master program. The generated list served as the sampling frame, and since it comprised a small number of patients, it was non-random, unlike a random sampling method, which involves probability and, therefore, elimination of some study subjects in the process. Consecutive sampling was used as the non-random sampling technique of choice, as it allowed moving from one patient on the list to the next, in sequence, calling each by telephone to assess their eligibility and traceability at the community level. All 36 participants met the inclusion criteria and were willing to participate; their physical addresses were confirmed, and they were contacted to schedule an interview at their premises. This process was performed until all eligible patients had been interviewed.

Data collection techniques

Data were collected between July and September 2025 at KSC, using structured interviews, to collect self-reported data, which involved asking respondents questions and providing multiple-choice options from which they chose the one that applied to them or their environment. Thus, the interview approach was sufficient for collecting all required data, and structured questionnaires were correspondingly the most suitable for capturing all responses generated in a given interview. However, structured interviews could not be used to validly collect data on whether or not a patient had shown up for their scheduled appendectomy. Therefore, another data collection method was employed to verify receipt of the appendectomy: document review. Patients who reported arriving for surgery at KSC had their claim cross-checked in the KSC patient database to confirm surgery reception. It was not until confirmation was made that a patient was categorized as a no-show or one who had shown up for their scheduled ambulatory appendectomy. The same was done for patients who reported not showing up for surgery, to avoid instances of patients denying receipt of the surgery. Once, a patient reported that they had not attended KSC for their procedure, but had the surgery done elsewhere; documentary evidence was still requested to confirm the claim.

All responses and/or data collected from structured interviews and document reviews were recorded on structured questionnaires, designed with closed-ended questions, which also had other merits, including the ability to capture quantifiable data. The questionnaire consisted of four sections: socio-demographic, no-show, intrapersonal, and institutional characteristic assessment questions (see Appendices). The questionnaire was designed by the principal investigator based on the findings (literature) from previous studies. Questions related to fear, affordability, and transportation were assessed using either Likert scale-type questions or nominal questions, which, for the sake of clarity, were accompanied by precise descriptions of their meanings. The questionnaire was electronic, and hence uploaded to the KoboToolbox (Harvard Humanitarian Initiative, Cambridge, USA) to consolidate all collected data and track it in real-time. With the Kobo Collect program (Harvard Humanitarian Initiative, Cambridge, USA) used, it was ensured that all mandatory questions were made mandatory and could not be skipped. This prevented the occurrence of missing responses and omissions in the final data set, which hence prevented possible deletion as a solution to handling missing data.

Questionnaire testing and validation

The principal investigator designed the questionnaire based on the literature and by adapting items from questionnaires used in previous studies that have assessed no-shows for appendectomy. The tool was further subjected to content-validated testing, which involved providing the questionnaire to four experts with experience in survey data collection. Some of these were clinicians at Kyabirwa Surgical Centre, familiar with scheduling appendectomy surgeries, and others were health researchers. Each of them was given the study objectives and a rating scale, with four ratings: 4 (very relevant), 3 (relevant), 2 (somewhat relevant), and 1 (not relevant). The mean number of items rated 4 or 3 was calculated by dividing the total number of those items rated by each expert by the number of experts. They were: 30 + 28 + 33 + 31 = 122 ÷ 4 = 31. The formula used to compute the content validity index (CV) was CVI = number of items rated 3 or 4 ÷ total number of items in the tool. That gave 31 ÷ 33 = 0.939, which fell within the range of 0.7 to 0.9, indicating that the tool had sufficient items to obtain all the data needed to achieve the study objectives, making it valid. 

Data analysis

The primary objective of this study was to determine the prevalence of no-shows for ambulatory appendectomy among patients undergoing ambulatory surgery. Therefore, the data collected for that objective were analyzed descriptively, that is, only in terms of frequencies and valid percentages. The findings at this level are presented as frequencies and percentages. Similar to the first objective, the data collected to analyze the drivers of no-shows for ambulatory appendectomy were analyzed descriptively, using both frequency distributions and cross-tabulation between the independent and outcome variables. Following this, all variables were subjected to bivariate analysis using a generalized linear model, specifically a log-binomial model, whose assumptions were fully met by the data obtained. The magnitude of the outcome for this study exceeded 10%, at which point the log-binomial model could generate accurate p-values [24]. The findings from the bivariate analysis were reported as crude prevalence ratios with 95% confidence intervals, as no confounder adjustment was made at that stage. The interest was in identifying variables to incorporate into a multivariate model; therefore, all variables with p-values <0.2 in the bivariate analysis were included in a multivariate log-binomial model, with potential confounders adjusted for. Variables that remained statistically significant at the 5% level after multivariate analysis were considered drivers of no-shows for ambulatory appendectomy. The findings at this level are presented as adjusted prevalence ratios, confidence intervals, and p-values.

Ethical considerations

This study was approved by the Mildmay Uganda Research Ethics Committee (MUREC) under the number MUREC-2024-394. The research permit to survey Uganda was obtained from the Uganda National Council of Science and Technology (UNCST) under the number HS6046ES. Permission to access patients at the Kyabirwa Surgical Center was obtained from the center's research committee. Each patient was first provided with details about the study, including its purpose, procedures, risks, benefits, and the ethical principles observed, including confidentiality and voluntary participation. Once each participant was furnished with that information, they were asked whether they were willing to participate in the study. Upon their consent, they were requested to sign a consent form as a sign of their participation. They were also told that all the information they provided would be handled with utmost confidentiality and anonymity, and that their names would not appear on their questionnaires. They only appended signatures on the consent forms or thumbprints. They were assured that none of the questionnaires they would complete would be accessed by anyone other than the principal investigator. All filled tools were stored in the principal investigator's personal locker, accessible only to her, and the computer used for data entry was password-protected with the password known only to the principal investigator. Even when a sampled adult was found in a large household, they were engaged in private interviews, assured that their participation in the study was voluntary, and assured of their choice to withdraw from the study.

## Results

Sociodemographic characteristics

Table 1 presents the findings from the assessment of the sociodemographic characteristics of the sampled patients. More than half of them (19, 52.8%) were female, while almost three-quarters (26, 72.2%) were aged 30 to 50 years. Nearly all the patients assessed were formally educated (34, 94.4%), although almost half (15, 44.1%) had received education up to the primary (upper) level. More than two-thirds of the respondents were currently officially married (n = 25, 69.4%), with the majority not in a cohabitation relationship (n = 8, 72.7%).

**Table 1:** Sociodemographic characteristics of the patients interviewed

Variable	n (%)
Sex of respondent	
Female	19 (52.8)
Male	17 (47.2)
Age	
30-50 years	26 (72.2
More than 50 years	10 (27.8)
Formally educated	
Yes	34 (94.4)
No	2 (5.6)
Education level	
Primary (Lower)	4 (11.8)
Primary (Upper)	15 (44.1)
Secondary	8 (23.5)
Post-secondary	7 (20.6)
Currently officially married	
Yes	25 (69.4)
No	11 (30.6)
Marital status: if not married	
Single/separated	3 (27.3)
Cohabiting	8 (72.7)

Ambulatory appendectomy shows status

Table 2 presents the findings from the assessment of show-up for ambulatory appendectomy among patients scheduled for that procedure at the KSC. It was shown that following the diagnosis of appendicitis at Kyabirwa Surgical Center and reception of a date on which the appendectomy had to be performed, the majority of the patients (26, 72.2%) returned to the center to undergo the procedure. However, not all of them underwent surgery at the center, although almost all underwent the procedure (n = 24, 92.3%). Nonetheless, exactly three-quarters of the patients (18, 75.0%) who underwent appendectomy received it from the KSC.

**Table 2:** Assessment of appendectomy uptake dynamics among patients sampled *Indicates the frequency that was used to compute appendectomy no-shows at KSC (Kyabirwa Surgical Center).

Variable	n (%)
When diagnosed with appendicitis at Kyabirwa Surgical Center, and given some medication and told to return on a particular date for an appendectomy to be done, I returned for the procedure.	
Yes	26 (72.2)
No	10 (27.8)
Appendectomy done	
Yes	24 (92.3)
No	2 (7.7)
Where was it done from	
KSC	18 (75.0)
Another facility	6 (25.0)

**Figure 1:**
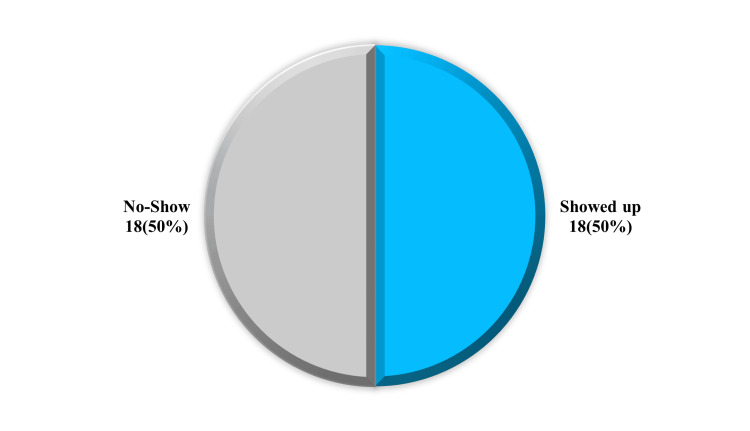
Prevalence of no-shows for ambulatory appendectomy

From the findings in Table 2, the prevalence of no-shows for ambulatory appendectomy among patients scheduled at Kyabirwa Surgical Center was computed and, as shown in Figure 1 below, found to be 50%.

Drivers of no-shows for ambulatory appendectomy

Table 3 shows results from the analysis of the drivers of no-show for ambulatory appendectomy at KSC, in which it is indicated that five factors were identified as significant. The prevalence of no-show for ambulatory appendectomy was less than 85% among patients who reported being able to afford the cost of appendectomy at the KSC without any hardships (aOR = 0.145, 95% CI = 0.024-0.859, p = 0.033). It was less than 87% among patients who had personal means of transport to the facility (aOR = 0.130, 95% CI = 0.019-0.876, p = 0.036) compared with those who had no such means. It was lower by 88% among patients who had access to means of transport to the facility (aOR = 0.120, 95% CI = 0.022-0.652, p = 0.014). However, the rate of no-show for appendectomy was 18 times higher among patients who perceived that the cost of appendectomy at the KSC was high (unaffordable) (aOR = 18.674, 95% CI = 1.424-24.824, p = 0.026). Patients who feared not waking up during appendectomy surgery (aPR = 26.059, 95% CI = 1.653-41.909, p = 0.021) were 26 times more likely to not show up for appendectomy than those who had no such fear.

**Table 3:** Drivers of no-shows for ambulatory appendectomy Statistical test used: log-binomial model for bivariate and multivariate analysis cPR = crude prevalence ratio; aPR = adjusted prevalence ratio

	Crude estimates		Adjusted estimates	
Variable	cPR (95% CI)	P value	aPR (95% CI)	P value
Was able to afford the cost of an appendectomy told at KSC, without any hardships				
Yes	0.160 (0.034 - 0.753)	0.020	0.145 (0.024 - 0.859)	0.033
No	1.000		1.000	
Had personal means of transport to the facility				
Yes	0.148 (0.034 - 0.636)	0.010	0.130 (0.019 - 0.876)	0.036
No	1.000		1.000	
Had access to means of transport to the facility				
Yes	0.385 (0.173 - 0.854)	0.010	0.120 (0.022 - 0.652)	0.014
No	1.000		1.000	
I feared that I might not wake up during the appendectomy surgery				
Strongly disagree	3.125 (0.278 - 35.157)	0.356	23.145 0.738 - 72.567)	0.074
Disagree	15.000 (0.663 - 39.548)	0.089	2.399 (0.095 - 60.845)	0.596
Undecided	2.500 (0.100 - 62.605)	0.577	26.059 (1.653 - 41.909)	0.021
Agree	20.000 (1.416 - 28.449)	0.027	20.000 (1.416 - 28.449)	
Strongly agree	1.000		1.000	
Perceived cost of appendectomy at Kyabirwa Surgical Center				
High (Unaffordable)	12.857 (1.290 - 18.144	0.029	14.019 (1.298 - 25.385)	0.030
Moderate (I could afford to pay most of the cost of an appendectomy)	2.400 (0.165 - 34.928)	0.522	2.491 (0.161 - 38.441)	0.513
Low (I could fully pay for the surgery)	1.000		1.000	

## Discussion

This study established that the prevalence of no-shows for ambulatory appendectomy among patients scheduled at the Kyabirwa Surgical Center was 50%. This finding suggests that only half of the patients scheduled for ambulatory appendectomy between 2022 and 2025 at Kyabirwa Surgical Center underwent those surgeries. This, therefore, generally leaves the other half with untreated appendicitis, which probably predisposes them to the development of a more severe disease and leads to a very poor quality of life characterized by severe pain. By the time this study was conducted (2025), the proportion of patients (50%) had not received treatment, even when some had been diagnosed with appendicitis and needed surgery two years prior. Thus, some of them are most likely at risk of developing complicated appendicitis [10, 17, 13], which requires more sophisticated intraoperative and postoperative interventions such as percutaneous drainage [13] and abdominal drainage (prophylactic) [14].

Thus, these patients might also be at risk of experiencing catastrophic health expenditures given the heightened costs of surgery that they will have to undergo in the future [14]. This reality might still be true, even when some choose to undergo surgeries at the Kyabirwa Surgical Center, whose ambulatory surgeries are known to be relatively cheaper than those provided at other Ugandan facilities. The effect of no-shows for ambulatory appendectomy was not only experienced at the patient level, but also at the institutional level. It has been estimated that every surgery no-show causes a loss of $196 for the institution that schedules the patient [25]. While this figure is not explicitly contextual to low- and middle-income countries, it clearly indicates that some institutional economic losses occur at surgical centers, consequent to no-shows [3]. This could, in the long run, affect surgical service delivery, compromise the quality of care, and perhaps impact the costs of surgery if the running costs of the surgery-providing facility are inflated. This could mean an increase in the significance of the cost barrier to surgery, even at ambulatory facilities such as the KSC, which may consequently lead to more cases of no-shows due to financial reasons.

No-shows for surgery are not a challenge at KSC alone; they have been documented among patients at other facilities as well. Few previous studies have quantified the prevalence of no-shows for ambulatory appendectomies; however, based on findings on surgery no-shows among patients scheduled for different surgical procedures, the prevalence of no-shows for ambulatory appendectomy at the KSC still ranks among the highest recorded in previous studies. For instance, it was higher than that previously reported by Dantas et al. (23%) [26] and Cohen-Yatziv (15%) [5] for Ear, Nose, and Throat (ENT) surgeries; and for orthopedic surgeries (6.12%) [27] and Emergency Department (<25%) [28] in South Korea. Those studies included patients who had been scheduled earlier for other forms of major surgery, including ear, nose, and throat surgeries, as well as orthopedic surgeries, which may have been perceived as beneficial in mitigating a very severe form of surgical disease, compared to those scheduled for appendectomy at KSC. Some of them had been given medication to take as part of the therapeutic management plan, and perhaps got some relief, which made them have low-risk perceptions, which could have led them, to some extent, not to show up for surgery.

On a positive note, however, the findings also showed that although some patients did not attend the scheduled ambulatory appendectomy at KSC, they proceeded to have it performed at other facilities (Table 2). The proportion of patients was 25%, which accounted for a total of 92.3% of the patients who underwent appendectomy, regardless of the institution from which they received it. Although this is a notable finding, it raises the question of why some patients scheduled for ambulatory appendectomy at KSC did not choose to undergo ambulatory surgery despite its provision of safe, cost-effective (cheaper) surgery, as Uganda’s only ambulatory surgical facility. Therefore, the study also included an assessment of no-show drivers for ambulatory appendectomy, upon which five drivers were identified.

One of the drivers was the perceived cost of appendectomy, for which the prevalence of no-show for appendectomy was 18 times higher among patients who perceived that the cost of appendectomy at KSC was high (unaffordable) than among those who reported that it was affordable. This finding relates to one of the greatest barriers to surgical access: its high cost, consistent with previous studies [29, 30]. The Kyabirwa Surgical Center, being a purely ambulatory surgical center, provides relatively affordable surgery, which is significantly subsidized in most cases. However, since the center was primarily set up to increase access to surgery among the rural poor, it comes as no surprise that even with surgery cost subsidization, some patients still perceived the cost of an appendectomy at KSC as unaffordable. That is, even though surgery costs at KSC are 50% less than those of other non-ambulatory hospitals in Uganda.

However, perceiving the cost of unaffordability can easily prevent one from having the surgery, in fear of experiencing catastrophic or impoverishing expenditures. What consequently ensues, in a bid to reduce pain and perhaps treat the disease, is the use of alternative medicines, including herbal medicines, which can further delay access to surgery, increasing the risk for complicated appendicitis. With the perceived high cost of ambulatory appendectomy as a driver of no-shows, it was not surprising that the no-show rate for ambulatory appendectomy was less than 85% among patients who reported being able to afford the appendectomy cost at KSC without any hardships. Owning or having access to transportation to a given health facility can lessen both the first and second delays of accessing healthcare. This alone can increase the odds of showing up for surgery, even for patients experiencing appendicitis-related pain, as they will be assured of reaching KSC using motorized means that are usually comfortable. Second, owning a means of transport is also a proxy indicator of socioeconomic status, typically indicating that one is not poor and can hence afford relatively subsidized surgery that requires out-of-pocket payments, such as that provided at the KSC.

Undoubtedly, the strongest driver of no-show for ambulatory appendectomy was tomophobia. The study established that patients who feared not waking up during appendectomy surgery showed a 26 times higher prevalence of not showing up for appendectomy compared to those who had no such fear. The fear of surgery is known to be a significant barrier to surgical access, given the heightened perceptions of susceptibility to intraoperative mortality and the severity of surgical outcomes. Such perceptions, even when misconceived, are sufficient to make one consider foregoing their scheduled surgery in a preconceived bid to stay alive and not succumb to death by a procedure meant to cure their ailment.

Limitations

This study inevitably included a small sample size, even following consideration of a four-year retrospective period, over which appendectomy patients had been scheduled. However, as justified in the sample size determination process, not all patients scheduled during the study period met eligibility criteria or were accessible for interview. Nonetheless, such a sample size came with a limitation of potentially leading to both type 1 and type 2 errors, due to low study power. To minimize type 2 error, we used a statistical model (log-binomial) whose assumptions were met by the magnitude of the study outcome. The second limitation that the study had was the use of consecutive sampling to sample patients, which, despite being the least biased of all non-random sampling methods, ultimately comes with some bias of limiting the generalizability of the findings. 

## Conclusions

The prevention of no-shows for appendectomy among patients at KSC is concerning; only half of the patients scheduled for that surgery appear at the center. One-quarter of them underwent surgery at other facilities, while the rest did not. This patient behavior is squarely linked to individual patient characteristics, largely tomophobia, and the perceived high cost of ambulatory appendectomies. In the future, the administration and staff of Kyabirwa Surgical Center may consider preoperatively educating and sensitizing all patients scheduled for ambulatory appendectomy or any ambulatory surgery about the comparatively lower cost of their ambulatory surgical procedures at Kyabirwa Surgical Center compared to other non-ambulatory facilities. This might alleviate the negative perceptions of the affordability of surgeries, such as appendectomies, which are at the center, hence reducing the no-show prevalence. While the Kyabirwa Surgical Center can not influence the ownership of transportation, efforts are made to educate patients booked for surgery about how they can access transportation at various locations along the route from different parts of the eastern central region, especially near the Kyabirwa Surgical Center. Preoperatively, sufficient education about the safety of ambulatory surgery and the demystification of myths surrounding anesthesia and its effects should be provided to all patients with tomophobia.

## Appendices

**Table 4:** Questionnaire

PART A: Socio-demographic characteristics							
Number			Question				Responses
1			Sex of respondent				Female Male
2			How old are you currently				…………………………………….
3			What religious denomination do you subscribe to?				Catholic Anglican Muslim Orthodox SDA Other………………….
4			Are you formally educated?				Yes No
5			If yes, what is your education level				Primary (Lower) Primary (Upper) Secondary (O level) Secondary (A level) Post-secondary Education
6			Are you currently officially married?				Yes No
7			If not, what is your marital status?				Cohabiting Single Separated
PART B: Show status for appendectomy							
8		When you were diagnosed with appendicitis at Kyabirwa Surgical Center, given some medication and told to return on a particular date for an appendectomy to be done. Did you return for the procedure?				Yes No	
9		If yes, was the appendectomy done?				Yes No	
10		Where was the appendectomy done from				Kyabirwa Surgical Center Jinja regional referral hospital Other (specific)……………………..	
PART C: Patient-related characteristics							
11	Appendicitis can be a fatal disease if not treated early			Strongly disagree Disagree Undecided Agree Strongly agree			
12	There are cases of appendicitis for which an appendectomy is the only effective treatment			Strongly disagree Disagree Undecided Agree Strongly agree			
13	Appendicitis cannot stop me from living my life; I can live with it			Strongly disagree Disagree Undecided Agree Strongly agree			
14	I think any person with appendicitis is at risk for complicated appendicitis			Strongly disagree Disagree Undecided Agree Strongly agree			
15	At the time you were booked for an appendectomy to be done at Kyabirwa, and told the cost of surgery, were you able to afford it, without any hardships?			Yes No			
16	At the time you were booked for an appendectomy to be done at Kyabirwa, did you have a personal means of transport to the facility?			Yes No			
17	At the time you were booked for an appendectomy to be done at Kyabirwa, did you have access to means of transport to the facility			Yes No			
18	I feared that I might not wake up during the appendectomy surgery.			Strongly disagree Disagree Undecided Agree Strongly agree			
19	I thought that the appendectomy might make me disabled			Strongly disagree Disagree Undecided Agree Strongly agree			
20	I feared that the appendectomy might turn me into a dependent			Strongly disagree Disagree Undecided Agree Strongly agree			
21	I didn’t think that an appendectomy could be safely done without serious side effects			Strongly disagree Disagree Undecided Agree Strongly agree			
22	For how long had you stayed with appendicitis at the time you came to Kyabirwa Surgical Center			………………………………………. ………………………………………..			
23	Did you use any traditional medicine for treating appendicitis after visiting Kyabirwa Surgical Center			Yes No			
24	Did you procure more modern medicine in an attempt to treat appendicitis, after scheduling for an appendectomy			Yes No			
25	I had trust that Kyabirwa Surgical Centre could carry out a safe appendectomy			Strongly disagree Disagree Undecided Agree Strongly agree			
Part D: Institutional characteristics							
Number	Question				Responses		
26	The distance between my home and Kyabirwa Surgical Center is quite long, so I couldn’t come for the surgery				Strongly disagree Disagree Undecided Agree Strongly agree		
27	How would you rate the quality of education about appendectomy that was given to you by the service providers at Kyabirwa Surgical Center				High (Was educated by staff, enough about appendectomy, its benefits, risks, safety and side effects) Moderate (Was somewhat educated by staff, enough about appendectomy, its benefits, risks, safety and side effects Low (Was not educated by staff, enough about appendectomy, its benefits, risks, safety and side effects		
28	Were you satisfied with the quality of patient-provider interaction that you had at KSC at the time of diagnosis of appendicitis?				Yes No		
29	Kyabirwa Surgical Center has enough qualified surgeons to handle an appendectomy				Strongly disagree Disagree Undecided Agree Strongly agree		
30	How would you rate the Healthcare provider attitude at the time of appendicitis diagnosis and booking for surgery				Positive Negative		
31	How would you rate the cost of appendectomy at Kyabirwa Surgical Center				High (Unaffordable) Moderate (I could afford to pay most of the cost of appendectomy) Low (I could fully pay for the surgery)		
32	Were you followed up by Kyabirwa staff to remind you about the impending appendectomy?				Yes No		
33	Did you understand the need for an appendectomy at Kyabirwa Surgical Center?				Yes No		
